# Psoriasis between Autoimmunity and Oxidative Stress: Changes Induced by Different Therapeutic Approaches

**DOI:** 10.1155/2022/2249834

**Published:** 2022-03-12

**Authors:** Marija V. Medovic, Vladimir Lj. Jakovljevic, Vladimir I. Zivkovic, Nevena S. Jeremic, Jovana N. Jeremic, Sergey B. Bolevich, Ana B. Ravic Nikolic, Vesna M. Milicic, Ivan M. Srejovic

**Affiliations:** ^1^Department of Dermatovenerology, University of Kragujevac, Faculty of Medical Sciences, Svetozara Markovica 69, 34000 Kragujevac, Serbia; ^2^University Clinical Center Kragujevac, Zmaj Jovina 30, 34000 Kragujevac, Serbia; ^3^Department of Physiology, University of Kragujevac, Faculty of Medical Sciences, Svetozara Markovica 69, 34000 Kragujevac, Serbia; ^4^I.M. Sechenov First Moscow State Medical University, Department of Human Pathophysiology, Moscow, Russian Federation, Trubetskaya Str. 2, 119992 Moscow, Russia; ^5^Department of Pharmacy, University of Kragujevac, Faculty of Medical Sciences, Svetozara Markovica 69, 34000 Kragujevac, Serbia

## Abstract

Psoriasis is defined as chronic, immune-mediated disease. Regardless of the development of new therapeutic approaches, the precise etiology of psoriasis remains unknown and speculative. The aim of this review was to systematize the results of previous research on the role of oxidative stress and aberrant immune response in the pathogenesis of psoriasis, as well as the impact of certain therapeutic modalities on the oxidative status in patients with psoriasis. Complex immune pathways of both the innate and adaptive immune systems appear to be major pathomechanisms in the development of psoriasis. Oxidative stress represents another important contributor to the pathophysiology of disease, and the redox imbalance in psoriasis has been reported in skin cells and, systemically, in plasma and blood cells, and more recently, also in saliva. Current immune model of psoriasis begins with activation of immune system in susceptible person by some environmental factor and loss of immune tolerance to psoriasis autoantigens. Increased production of IL-17 appears to be the most prominent role in psoriasis pathogenesis, while IL-23 is recognized as master regulator in psoriasis having a specific role in cross bridging the production of IL-17 by innate and acquired immunity. Other proinflammatory cytokines, including IFN-*γ*, TNF-*α*, IL-1*β*, IL-6, IL-22, IL-26, IL-29, or IL-36, have also been reported to play important roles in the development of psoriasis. Oxidative stress can promote inflammation through several signaling pathways. The most noticeable and most powerful antioxidative effects exert various biologics compared to more convenient therapeutic modalities, such as methotrexate or phototherapy. The complex interaction of redox, immune, and inflammatory signaling pathways should be focused on further researches tackling the pathophysiology of psoriasis, while antioxidative supplementation could be the solution in some refractory cases of the disease.

## 1. Introduction

Psoriasis was firstly described in detail by Robert Willan, founder of dermatology as a medical specialty [1]. The prevalence of psoriasis varies from 0.51% to 11.43% in adults, making psoriasis one of the most important global health problems [2]. According to the World Health Organization, psoriasis is classified as one of the most serious noninfectious diseases, due to complications that develop during the course of the disease and affection of multiple organ systems [3, 4]. Psoriasis is defined as chronic, inflammatory, recurrent, incurable, and noncontagious disease, characterized by sharply demarcated erythematous skin lesions with overlying silver hyperkeratotic plaques, accompanied by systemic manifestations [3].

### 1.1. Clinical Presentation of Psoriasis

**Figure 1:**
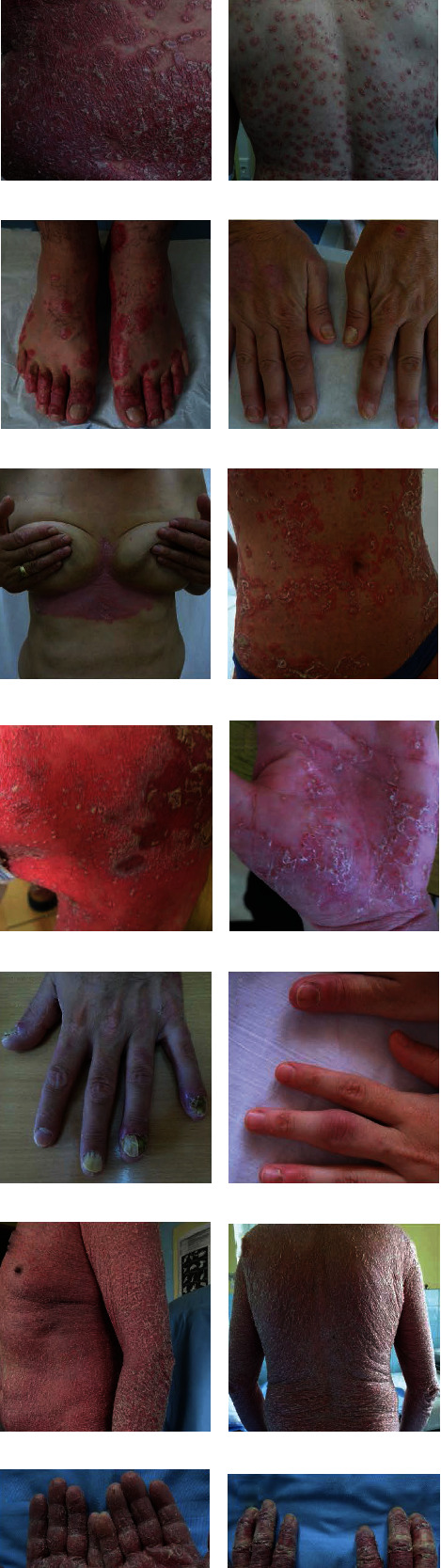
Clinical manifestations of psoriasis. Psoriasis vulgaris manifested through typical erythematous plaques with silvery scales of various sizes from guttate lesions through nummular to giant lumbar plaque (a). Psoriasis vulgaris and guttate psoriasis (b, d). Generalized psoriasis (c, h, i). Inverse psoriasis (g). Psoriatic lesions on the feet and hands (e, f, j, k), including psoriatic nail dystrophy (f, k) and psoriatic arthritis (l). Erythrodermic psoriasis, erythema, and squamous cover 90% of the skin (m, n, o, p). Pictures presented are part of the private collection of Vesna M. Milicic and Ana B. Ravic Nikolic.

The most distinctive characteristic of psoriasis are well-defined, symmetric, raised skin lesions most commonly located on the knees, elbows, scalp, and trunk [5]. Such clinical presentation is a characteristic for plaque psoriasis or psoriasis vulgaris, the most common form of psoriasis, but psoriasis also may appear as guttate, pustular (von Zumbusch psoriasis) or erythrodermic psoriasis (Figure 1). Skin changes are usually accompanied by pruritus, itching, pain, cracking, bleeding, and flaking of the skin. Furthermore, psoriasis is recognized as a risk factor for many pathological conditions, including cardiovascular diseases, gastrointestinal disorders, malignant tumors, infections, and mood disorders [6]. The most common comorbidity of psoriasis is psoriatic arthritis, usually defined as heterogeneous inflammatory arthritis which affects joints and entheseal tissues [7]. Such interconnection of psoriasis and the variety of comorbidities probably arises from, on the one hand, complex etiological and pathophysiological basis of the disease, and on the other hand, the fact that psoriasis remains unrecognized and untreated for a long period [8, 9].

Psoriasis vulgaris is generally equally present among the sexes, but it develops somewhat earlier in women than in men [3]. The severity of psoriasis is usually classified using Psoriasis Area and Severity Index (PASI) and Body Surface Area (BSA) [10]. Due to the common association of psoriasis and psoriatic arthritis, several screening questionnaires are performed for early recognition of psoriatic arthritis, such as Psoriasis and Arthritis Screening Questionnaire (PASQ), Psoriasis Epidemiology Screening Tool (PEST), and Toronto Psoriatic Arthritis Screen (ToPAS) [11].

### 1.2. Main Pathophysiological Features of Psoriasis

Psoriasis is defined as chronic, immune-mediated disease. Regardless of the development of new therapeutic approaches, the precise etiology of psoriasis remains unknown and speculative. Complex immune pathways of both the innate and adaptive immune systems appear to be pathophysiological basis in the development of psoriasis [12, 13]. Furthermore, epidemiological investigations indicated the importance of genetic component in the development of psoriasis [14]. Firstly, it was noticed that psoriasis concordance is higher in monozygotic twins [15]. Recent genetic studies identified almost 60 psoriasis susceptibility loci which could interfere with the development of psoriasis [14]. Various environmental factors, such as stress, mechanical trauma, and streptococcal infections are considered to trigger and not cause the disease. In predisposed persons, various factors may provoke the onset of disease or exacerbation of existing symptoms.

Due to the complex pathophysiological mechanisms that affect various tissues and organs, psoriasis may be defined as a systemic disorder with the predominant skin representation. Cutaneous psoriatic manifestations occur as a result of disruption of skin homeostasis due to immune-mediated aberrant differentiation of keratinocytes [12]. Interleukin- (IL-) 23 and IL-17 are recognized as key immune mediators that mediate not only the development of skin lesions but also the occurrence of psoriasis-associated comorbidities [3]. Skin lesions, followed by extracutaneous comorbidities and chronic course of the disease, significantly affect the quality of life of the psoriasis-suffering patients resulting in the development of anxiety and depression [16].

Psoriasis is a chronic, incurable disease, so treatment of psoriasis can reduce skin lesions, but not provide a complete cure. The psoriatic therapy varies depending on the surface of affected skin—PASI [17]. The most effective therapeutics in psoriasis treatment are biologic therapies, recently developed drugs that target some immune pathway. Drugs from this group act in different ways and target diverse cytokines: tumor necrosis factor *α* (TNF-*α*) (adalimumab, certolizumab pegol, etanercept, and infliximab), IL-12/IL-23p40 (ustekinumab), IL-17A (ixekizumab, secukinumab), IL-17 receptor (brodalumab), and IL-23p19 (guselkumab, risankizumab, and tildrakizumab) [18] (Table 1). Biologics are usually used in severe cases of psoriasis. Mild disease is treated with topical preparations combined with phototherapy, and moderate psoriasis is treated with immunomodulatory therapy.

Reactive oxygen species (ROS) represent important regulators of immune response [19]. The imbalance in production and elimination of ROS results in oxidative stress and, consequently, oxidative damage of various cellular structures. Proinflammatory processes involved in the development of autoimmune disorders are combined with increased production of ROS and oxidative stress. Redox imbalance in psoriasis exists both in skin cells and, systemically, in plasma and blood cells [20]. Thus, ROS and oxidative stress appear to be an important step in the psoriasis pathophysiological cascade. The aim of this review is to show the relationship between redox balance and applied therapy in patients suffering from psoriasis.

### 1.3. Autoimmune Nature of Psoriasis

**Figure 2:**
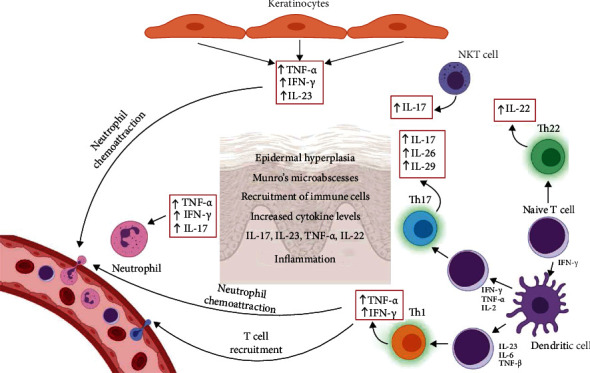
Immune response in psoriasis. Complex interaction of various parts of innate (dendritic cells, NKT cells, and neutrophils) and acquired immunity (T cells) in pathophysiology of psoriasis. IFN: interferon; IL: interleukin; NKT: natural killer T; Th: T-helper cell; TGF: transforming growth factor; TNF: tumour necrosis factor.

The main feature of autoimmune disorders is destruction of healthy tissues by the host immune system upon misidentification and recognition of its own tissue structures as foreign. The results of the immunological studies in the recent decades showed involvement of both innate and acquired immunity and importance of T cells in pathogenesis of psoriasis [21–23]. T helper (Th) cells are classified as Th1 cells, which predominantly secrete TNF-*α*, interferon- (IFN-) *γ*, and IL-2, and Th2 cells, which secrete IL-4, IL-5, IL-10, and IL-13 [24]. Determination of naïve T cells toward Th1 or Th2 subpopulation depends on the stimulation by IL-12, which mediates Th1 differentiation, or IL-2, which mediates Th2 differentiation [25]. Due to increased levels of TNF-*α* and IL-12 in psoriatic lesions, psoriasis is defined as Th1-mediated disease [26]. IL-23 is another important cytokine in the development of psoriasis because it shifts the differentiation of naïve T cells toward proinflammatory Th17 cells [27]. These findings are the backbone of the development of new therapeutic approaches such as biologic therapies. Apart from T cells, as representatives of acquired immunity, neutrophils, dendritic antigen presenting cells (APCs), and Natural Killer T (NKT) cells in pathogenesis of psoriasis play an important role [28]. One of the first described histopathological hallmarks of psoriasis was Munro's microabscesses containing neutrophils [29]. Increased neutrophil activity in psoriatic plaques results in increased ROS production [30] (Figure 2, Table 2). ROS act as stimulators of dendritic APCs to present antigens to T cells, which further stimulate proliferation of keratinocytes [29]. ROS also have a role of second messengers in inflammatory signaling cascades involving activation of mitogen-activated protein kinase (MAPK), nuclear factor-kappa B (NF-*κ*B). NKT cells encompass heterologous cells which share some features of the natural killer (NK) and T cells. In psoriatic patients, NKT cells interact with other components of immune system, thereby supporting the creation of proinflammatory environment. Subset of NKT cells, NKT17 cells, can produce IL-17, and they are found in many tissues, among others in the skin, but their role in the pathogenesis of psoriasis is not fully understood [31, 32].

Keratinocytes play an important role in physiological orchestration of innate and acquired immune responses in the skin due to various pathological stimuli. Injured keratinocytes may produce TNF-*α* and IFN-*γ* [13]. TNF-*α* induces increased neutrophil accumulation in injured skin, and IFN-*γ* increases recruitment of Th1 cells. Psoriatic plaques contain increased number of T lymphocytes which produce IFN-*γ*, IL-17, and IL-22, labeled as Th1, Th17, and Th22 cells, respectively [13].

Dendritic APCs may have a crucial role in pathogenesis of psoriasis due to the activation of T cells and production of various proinflammatory cytokines. Population of skin dendritic cells include epidermal dendritic cells (Langerhans cells) and dermal dendritic cells (myeloid and plasmacytoid dendritic cells). CD11c is recognized as the correct marker of myeloid dendritic cells, while blood dendritic cell antigen (BDCA) is used for identification of different human subsets of dendritic cells [33]. Myeloid CD11c^+^BDCA-1^–^ dermal dendritic cells are recognized as proinflammatory dendritic cells which induce activation and clonal expansion of CD4^+^ and CD8^+^ T cells and their stimulation in the production of IFN-*γ*, IL-17, and IL-22 [34]. Furthermore, it was shown that content of CD11c^+^BDCA-1^–^ dermal dendritic cells is increased in psoriatic lesion, but their number is significantly reduced upon effective therapeutic approach [35].

The current immune model of psoriasis begins with the activation of immune system in susceptible persons by some environmental factor and a loss of immune tolerance to psoriasis autoantigens [34]. TNF-*α* and IFN-*γ*, secreted by dendritic cells, induce polarization and expansion of IL-17 and IL-22 secreting T cells (Th17 and Th22 cells), resulting in significant increase of the IL-17 and IL-22 production (Figure 2, Table 2). IL-17 is recognized as the key cytokine in the development of psoriasis. There are six isoforms of IL-17 (IL-17A–IL-17F), whereby IL-17A has the most prominent role in psoriasis pathogenesis [36]. Th17 cells, besides IL-17, produce TNF-*α*, IL-26, and IL-29. IL-17A, alone or synergistically with TNF-*α*, induces the release of proinflammatory molecules from keratinocytes and enhances aberrant proliferation of keratinocytes leading to epidermal hyperplasia. Increased production of IL-26 and IL-29 by Th17 stimulate further release of proinflammatory mediators which recruit Th1 cells into psoriatic skin lesions [37, 38]. Increased production of IL-17 results in increased secretion of IL-19, IL-22, and IL-36 which also contribute to the development of epidermal hyperplasia [39, 40]. IL-22 in psoriatic lesions is secreted not only by CD4^+^ and CD8^+^ T cells known as Th22 and Tc22 cells but also by Th17, mast cells, and others [41]. IL-22 enhances migration of keratinocytes, increases epidermal thickness, decreases keratinocyte differentiation, and stimulates secretion of various molecules which act as chemokines, neutrophil chemoattractants [41]. IL-22 proinflammatory action is weaker than IL-17, but IL-22 mainly acts synergistically with IL-17 and TNF-*α*. Dendritic cells also secrete IL-23 which acts via the IL-23 receptor located on naïve T cells and promotes their differentiation into Th17 [42]. IL-23 belongs to the IL-12 cytokine family. It is a composite cytokine containing two subunits IL-23p19 and IL-12p40 [43]. Acting together with TNF-*α*, IL-1*β*, and IL-6, IL-23 stimulates the differentiation of Th17 and Tc17 cells and the conversion of regulatory T cells (T_reg_) into Th17 cells [44]. After the differentiation of naïve T cells to Th17 cells due to various stimuli such as transforming growth factor (TGF)-*β* and IL-6, the presence of IL-23 is necessary to maintain the Th17 phenotype [25, 45]. Binding of IL-23 to its receptor on Th17 cells initiates signaling pathway, which results in the facilitation of IL-17 expression and increased levels of IL-17A in plasma [46]. Besides the action of IL-23 on T cells and induction of them to produce IL-17, thus provoking the inflammatory autoimmune response (acquired immunity), it can also stimulate the production of IL-17 by NK cells and neutrophils (innate immunity) [47, 48]. Thus, IL-23 is recognized as a master regulator in psoriasis having a specific role in cross bridging the production of IL-17 by innate and acquired immunity. The administration of IL-23 in mice induced epidermal hyperplasia and increased expression of both IL-17A and IL-22 [49]. Psoriasis is defined as Th17-mediated disease, but results of a growing number of studies indicate the central role of IL-23 in the development of psoriasis due to its effects on sustention of cytotoxic Th17 cells and production of IL-17 and IL-22 (Figure 2, Table 2). Altogether, various parts of acquired and innate immune system create complex signaling pathways in the development of psoriasis.

### 1.4. Oxidative Stress in Pathogenesis of Psoriasis

Oxidative stress is usually defined as an imbalance between the production of ROS or reactive nitrogen species (RNS) and antioxidative capacity. Decreased antioxidative ability may be the consequence of decreased activity of antioxidative enzymes (such as superoxide dismutase (SOD), catalase (CAT), and glutathione peroxidase (GPx)) or/and decreased concentration of scavenging antioxidants, both endogenous (reduced glutathione (GSH)) and exogenous (vitamin C, vitamin E, carotenoids, and others). At low, physiological concentrations, ROS/RNS have important roles as signaling molecules in regulatory cascades of different biological processes, but excess ROS/RNS and consequent oxidative stress induce oxidation of various cellular structures (DNA, lipids, and proteins) leading to cell death [50, 51].

**Figure 3:**
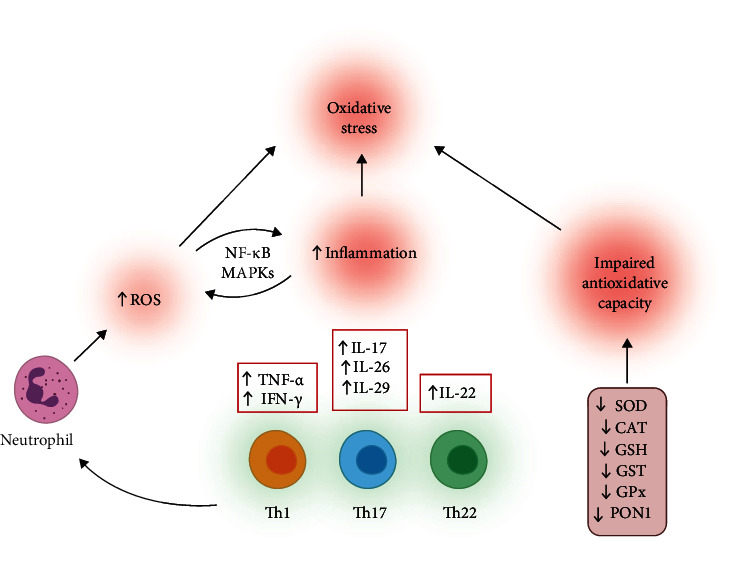
Mechanisms involved in increasing oxidative stress in psoriasis and interactions between oxidative stress and inflammation. IFN: interferon; IL: interleukin; MAPKs: mitogen-activated protein kinases, NF-*κ*B: nuclear factor kappa B; ROS: reactive oxygen species; Th: T-helper cell; TNF: tumour necrosis factor.

The skin and thus keratinocytes are continuously exposed to various external stressful stimuli, including ultraviolet (UV) radiation of the sun, and oxygen from the air. It is assumed that more than 50% of skin damage induced by UV radiation is mediated by ROS/RNS [52]. Furthermore, different toxic substances, as well as their metabolites, directly or indirectly initiate the production of various prooxidative molecules in keratinocytes [53]. Oxidative stress can promote inflammation through several signaling pathways including NF-*κ*B, mitogen-activated protein kinases (MAPKs), and STAT3 (Signal Transducer and Activator of Transcription 3) [54]. MAPKs represent a family of serine-threonine protein kinases encompassing several members: extracellular signal-regulated kinases (ERKs), c-Jun N-terminal kinases (JNKs), and the p38 MAPKs [55]. Increased presence of ROS and impaired antioxidative potential directly induce increased activation of NF-*κ*B [56]. Immunohistochemical analysis of psoriatic skin lesions showed increased levels of phosphorylated ERK1/2 and p38 MAPK [57, 58]. It was shown that propranolol induced psoriasis-like skin inflammation through increase of oxidative stress as well as NF-*κ*B and MAPK p38 activation and subsequent secretion of IL-23 [59]. Results of this research confirmed the crucial role of oxidative stress in IL-23/IL-17 axis of Th17-related psoriasis-like skin inflammation (Figure 3, Table 3). ROS produced in the skin also act as chemoattractant for neutrophils and, furthermore, increased number of neutrophils combined with high levels of ROS may result in the activation of neutrophils and further increase of ROS production [60, 61]. Augmented inflammatory response further facilitates production of ROS and decreases already weakened antioxidative capacity which makes psoriasis a chronic inflammatory disease [62].

The nuclear factor erythroid 2-related factor 2 (Nrf2) is redox-sensitive transcription factor involved in the regulation of keratinocyte proliferation and expression of keratin [63]. There are ambiguous data regarding the role of Nrf2 in pathogenesis of psoriasis. Lee et al. pointed out increased oxidative damage in psoriatic skin lesions combined with decreased expression of Nrf2, while dimethyl fumarate, as an Nrf2 activator, upregulated Nrf2 levels in HaCaT keratinocyte cell line and promoted growth inhibition and apoptosis [64]. The authors assumed that the increased production of ROS and oxidative stress interfere with dysregulation of the Nrf2 signaling cascade. Another study also showed a decrease of ROS and increase of nuclear accumulation of Nrf2 after the application of antioxidant, followed by the reduction of vascular endothelial growth factor (VEGF) and the reduction of keratinocyte proliferation in a similar experimental model [65]. On the other hand, Yang et al. presented increased nuclear-localized Nrf2 in psoriatic epidermis compared to normal skin [63]. Increased expression of Nrf2 was linked with higher expression of psoriasis-related keratins K6, K16, and K17. Furthermore, it was shown that IL-17 and IL-22 enhance the proliferation of psoriasis-related keratins via Nrf2 signaling. It appears that redox signaling and Nrf2 poses divergent functions in the development of psoriasis, probably due to the cellular localization of Nrf2; however, many questions are to be answered further.

Several studies indicated an increased risk for the psoriasis occurrence in persons with polymorphisms of specific genes that are related to the regulation of redox balance (Figure 3, Table 3). Asefi et al. indicated an increased risk for the development of psoriasis in persons bearing 55 M allele for paraoxonase 1 (PON1) [66]. PON1 is hydrolytic enzyme bound to high-density lipoprotein (HDL), able to break down lipid peroxides. It is assumed that enzymatic activity of PON1 is crucial for protective effect of HDL. The PON1 55 M allele in psoriatic patients was found to be associated with higher malondialdehyde (MDA) levels, apolipoprotein B, and lipoprotein (a), suggesting interference of oxidative stress and disturbances in lipid metabolism in the pathogenesis of psoriasis [66]. Another study also revealed lower PON1 activity in psoriasis-suffering patients due to PON1 polymorphism related to decreased antioxidative activity and different lipid levels [67]. Glutathione S-transferases (GSTs) are a group of enzymes involved in catalytic regulation of the conjugation of GSH to various substrates, thus providing protection against various detrimental factors including oxidative stress and inflammation. Some polymorphisms of GST genes were significantly more common in patients with psoriasis compared to the healthy population [68]. Furthermore, null polymorphisms for GSTs were related to increased sensitivity psoralen-ultraviolet A (PUVA) photochemotherapy [69]. The activity of SOD also appears to be an important factor in the development of psoriasis. Knock out (KO) of extracellular SOD in mice induced more intense IL-23-mediated skin inflammation characterized with elevated accumulation of CD4^+^ T cells, CD11b^+^ macrophages, and CD11c^+^ dendritic cells accompanied by increased expression of proinflammatory cytokines [70]. Naïve CD4^+^ T cells were more differentiated into the Th17 cell in extracellular SOD KO mice compared to the wild type (WT) controls. The previous study showed decreased activity of SOD and CAT in erythrocytes of psoriatic patients [71]. Levels of MDA were higher in these patients, combined with decreased activity of antioxidative enzymes. An interplay between various components of the immune system, ROS/RNS, and antioxidative system creates intertwined signaling pathways.

Analyzing the differences in various pro- and antioxidants in stimulated and unstimulated saliva of psoriatic patients and healthy individuals, it was shown that several prooxidative markers were increased in patients with plaque psoriasis [72]. Contrary to the previous study [71], antioxidative enzymes were significantly higher not only in saliva but also in erythrocytes, of psoriatic patients compared to healthy control [72]. The same authors also showed significantly higher levels of TNF-*α*, IFN-*γ*, and IL-2, nitric oxide (NO), and nitrotyrosine in saliva of psoriatic patients compared to healthy individuals [73]. Furthermore, it was shown that various inflammation-related proteins and microbiota were changed in saliva of psoriatic patients [74, 75]. Thus, oxidative stress biomarkers, such as total oxidative status or oxidative stress index, as well as inflammatory markers could be considered as diagnostic tool in psoriasis.

### 1.5. Therapeutic Approaches in Psoriasis

Various treatments have been used for psoriasis patients, from topical medications that contain corticosteroids, retinoid derivatives, synthetic vitamin D3 analogues, tar, or anthralin to systemic drugs with a different mechanism of action. However, all therapeutic modalities have transient curative effects, and it is also difficult to predict the occurrence of exacerbations and determine which drug delays their occurrence most effectively. Most of the therapeutics used in psoriasis curation are not suitable for a long-term use due to serious side effects and high costs [54]. Until recently, drugs such as methotrexate, acitretin, cyclosporine, dexamethasone, and salicylic acid were most commonly used in psoriasis treatment. Many of these drugs have limited clinical efficacy due to different shortcomings including low absorption capacity, inconsistent drug release, low target tissue selectivity, and retention of drug molecules in the target tissue, as well as various adverse reactions [76].

Groundbreaking shifts in psoriasis therapy were achieved with the introduction of drugs that target key immune cascades in the pathogenesis of psoriasis. Biologic therapies or biologics represent progress in the treatment of psoriatic patients in the last decade, due to improved efficacy and tolerability of the drugs and consequently improved quality of life of the patients [77]. Novel biological drugs, which target TNF-*α*, the p40 subunit of IL-12 and IL-23, or IL-17 receptors, are effective in treating psoriasis and reducing the PASI score [78] (Table 1). Interestingly, inhibition of IL-1, IL-6, or IFN-*γ* failed to achieve a significant clinical effect in psoriasis [79]. TNF-*α* inhibitors include etanercept, infliximab, adalimumab, certolizumab pegol, and golimumab [80]. Inhibitors of IL-17 include secukinumab, ixekizumab, and bimekizumab, while brodalumab is inhibitor of IL-17 receptor [80]. Inhibition of 12p40 subunit, which is common for IL-12 and IL-23, represents the mechanism of action of ustekinumab and briakinumab, while guselkumab, tildrakizumab, and risankizumab are specific human antibodies targeting the p19 subunit of IL-23, thus blocking the biologic activity of IL-23 [80, 81].

Due to considerable prevalence of psoriasis in general population, as well as comorbidities related to psoriasis, new therapeutic modalities are constantly being investigated. In patients with severe cases of psoriasis in which established therapeutic procedures did not give desired results, the application of cell therapy is being examined, which includes the hematopoietic stem cell transplantation (HSCT) and mesenchymal stromal cell (MSC) [82, 83]. In addition to the fact that mesenchymal cell therapy brings the possibility of a complete recovery of patients with psoriasis, there are also a number of possible adverse effects that reduce the aspiration for their use. The possible adverse effects of cell therapy include neoplastic proliferation, graft versus host reaction, localized skin reactions, and a lack of efficacy [82]. Botulinum toxin has also been shown to be an effective drug in treatment of plaque-type psoriasis [84]. Hydrogen sulfide (H_2_S) is recognized as important mediator of various physiological processes and a crucial antioxidative molecule. Due to the disturbed redox balance in psoriasis, therapeutic value of H_2_S should be investigated [85]. Antioxidative support to applied antipsoriatic drugs could be an important factor in the reduction of keratinocyte proliferation and remission of the disease [65, 86, 87].

### 1.6. Effects of Biologic Therapeutics on Oxidative Stress

#### 1.6.1. Effects of IL-12 and IL-23 Inhibitors on Oxidative Stress

Ustekinumab and briakinumab bind to the p40 subunit which is common to IL-12 and IL-23 preventing the interaction of these cytokines with their receptors. Both drugs are fully human monoclonal antibodies. Ustekinumab was approved by the US Food and Drug Administration (FDA) and the European Medicines Agency (EMA) in 2009 for treatment of mild to severe cases of psoriasis, while all clinical trials for briakinumab were discontinued due to the increased risk for myocardial infarction, cerebrovascular accident, and cardiac death [88, 89].

**Figure 4:**
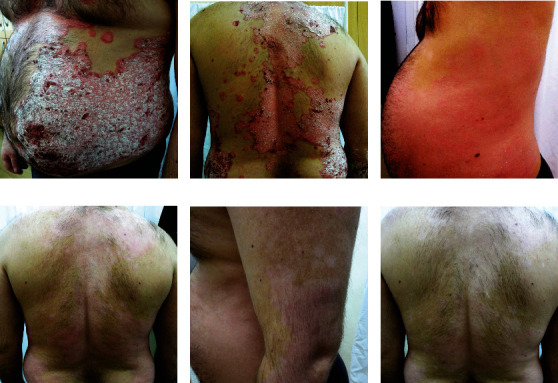
Clinical efficacy of IL-12 and IL-23 blockade (ustekinumab). Patient before starting therapy (a, b), after 8 weeks from the introduction of the IL-12/IL-23 blocker (c, d), and after 16 weeks after the introduction of the IL-12/IL-23 blocker (e, f). Pictures presented are part of the private collection of Vesna M. Milicic and Ana B. Ravic Nikolic.

There are very limited data regarding the effects of ustekinumab on redox balance in patients with psoriasis. In a randomized clinical trial, the effects of ustekinumab (IL-12/IL-23 inhibitor), etanercept (TNF-*α* inhibitor), or cyclosporine were compared regarding the heart function and oxidative stress in patients with psoriasis [90]. After the four-month treatment, the MDA level in patients treated with ustekinumab was significantly decreased compared to TNF-*α* inhibition, where it was unchanged, or cyclosporine, where it was even increased. Another antioxidative effect of anti-IL-23 antibody was shown in experimental cerebral ischemia [91]. The application of anti-IL-23 antibody decreased the production of ROS and MDA levels in the serum and brain. The antioxidative mechanism achieved by blocking of IL-23 in this experimental model involved targeting the immune specific Janus kinase 2- (JAK2-) STAT3 pathway. Clinical efficacy of IL-12 and IL-23 blockade (ustekinumab) is presented in Figure 4.

#### 1.6.2. Effects of IL-17A Inhibitors on Oxidative Stress

**Figure 5:**
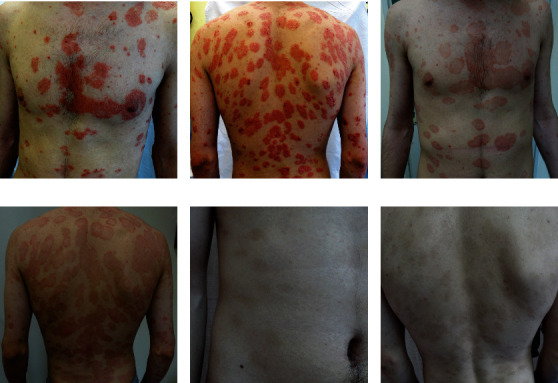
Clinical efficacy of IL-17A blockade (secukinumab). Patient before starting therapy (a, b), after 8 weeks from the introduction of the IL-17A blocker (c, d), and after 16 weeks after the introduction of the IL-17A blocker (e, f). Pictures presented are part of the private collection of Vesna M. Milicic and Ana B. Ravic Nikolic.

Antioxidative effect of IL-17 inhibition was first reported in a 42-year-old Caucasian woman suffering from plaque psoriasis [92]. One month after secukinumab therapy, plasma levels of lipid measured as thiobarbituric acid reactive substances (TBARS) were significantly decreased, while total antioxidant capacity was significantly improved. It was also noticed that neutrophils, monocytes, and lymphocytes decreased the production of ROS. In three murine experimental models of psoriasis-like skin disease, levels of IL-17A correlated with the severity of the disease and vascular dysfunction [93]. Treatment with anti-IL-17A antibody efficiently eradicated cutaneous lesions and decreased peripheral oxidative stress in two (CD11c-IL-17A^ind/ind^ mice and mice with imiquimod induced psoriasis) assessed the models of psoriasis. Interestingly, in the experimental model of severe psoriasis using K14-IL-17A^ind/+^ mice, neither skin lesions nor peripheral oxidative stress was improved [93]. Another study compared the effects of secukinumab (IL-17A inhibitor), cyclosporine, or methotrexate (MTX) treatment on the left ventricular function and oxidative stress in patients with psoriasis [94]. Secukinumab exerted most beneficial effects on the improvement of the left ventricular function and the decrease of MDA and protein carbonyl as markers of oxidative stress. On the other hand, MTX did not change the measured parameters for oxidative stress, while cyclosporine even caused them to increase [90]. The results of the Eding et al. showed that MutT Homolog 1 (MTH1) levels were increased in skin lesions of imiquimod-induced psoriatic mice [95]. Inhibition of MTH1 resulted in both decrease of ROS and oxidative stress and IL-17-mediated inflammation. Assessing the effect of IL-17A overexpression in CD4-IL-17A^ind/+^ mice, it was shown that IL-17A overexpression was accompanied by increased peripheral ROS/RNS levels, as well as increased ROS/RNS production by spleen CD11b^+^ cells [97]. Increased production of IL-17A, followed by an increase in oxidative stress, resulted in endothelial dysfunction and vascular damage. Clinical efficiency of IL-17 inhibition (secukinumab) is shown in Figure 5.

#### 1.6.3. Effects of TNF*α* Blockers on Oxidative Stress

TNF-*α* alone does not elicit a significant response to keratinocytes. However, in combination with IL-17A and other cytokines, TNF-*α* is a significant element of the cytokine milieu in psoriasis. Powerful synergism between TNF-*α* and IL-17A enhances the effects of IL-17A. In addition, it leads to increased expression of IL-17R by keratinocytes [97]. TNF-*α* inhibitors (etanercept, adalimumab, and infliximab) are human fusion proteins used in treatment psoriasis. Aiming to investigate the effects of infliximab, as TNF-*α* blocker, on redox balance of psoriatic patients, Barygina and coworkers have measured various oxidative stress biomarkers in plasma and white blood cells [98]. After six-month infliximab treatment, plasma levels of MDA and protein carbonyl content were decreased, while the activity of NADPH oxidase was decreased compared to untreated patients. The most significant limitation of this study is the very small number of patients, given that only 29 patients in total were included. In another study dealing with the role of etanercept, another TNF-*α* inhibitor, the authors measured plasma, total antioxidative capacity, and PON1 activity [99]. Etanercept application for 24 weeks significantly improved total antioxidative capacity and PON1 activity combined with reduction of inflammatory markers (C-reactive protein). Oxidative stress was also reduced by etanercept in experimental peritonitis [100]. Inhibition of TNF-*α* by adalimumab, human monoclonal antibody against TNF-*α*, showed significant reduction of oxidative stress in experimental model of vascular dementia [100]. Comparing the effects of ustekinumab, etanercept, or cyclosporine on oxidative stress in psoriasis-suffering patients, ustekinumab exerted most powerful antioxidative effect [92].

Overall, it can be concluded that all biologics exert antioxidative capacity but novel study comparing the antioxidative effects of biologics of different mechanism of action should offer new insights into interfering networks of oxidative stress, immune response, and inflammation.

### 1.7. Effects of Immunomodulatory Therapy on Oxidative Stress

Methotrexate (MTX) is a well-established drug for systemic treatment of psoriasis due to its antiproliferative, immunosuppressive, and anti-inflammatory properties. Its exact underlining mechanisms are not fully elucidated [102]. The antiproliferative effects of MTX are based on inhibitory effect on folate-dependent enzymes, such as dihydrofolate reductase, resulting in decreased synthesis of DNA and reduced cell proliferation [103]. Immunosuppressive and anti-inflammatory ability of MTX are a consequence of increased adenosine release, decreased release of proinflammatory cytokines (TNF-*α* and IL-1), favored apoptosis of activated T cells, and decreased chemotaxis of neutrophils [103].

A twelve-week MTX treatment in psoriatic patients induced augmentation of oxidative stress, reflected through increased levels of MDA and reduction of nitrate/nitrite, SOD, and CAT activity and TAS in the plasma [104]. The authors proposed that increased ROS and decreased NO production, combined with increased caspase-3 expression, represent the mechanism of MTX-mediated induction of apoptosis. Experimental study in an isolated hepatocytes rat confirmed prooxidative effects of MTX [105]. Antioxidants decreased ROS availability and reduced MTX-induced cytotoxicity. MTX also induced mitochondria swelling, decrease of ATP and GSH amount, and release of cytochrome c. The assessment of expression of isoenzymes of GST and cytochrome (CYP) families showed that expression was increased in patients with psoriasis compared to healthy controls [106]. After 12-week MTX treatment, the expression of these enzymes, important in regulation of redox balance, did not change significantly, although there was marked clinical improvement. Prooxidative effects of MTX were shown in other tissues and organs. In the MTX treated rats, the levels of GSH, SOD, and CAT testicular were significantly decreased, combined with increased DNA and tissue damage [107]. MTX also showed prooxidative features in liver and neural tissues due to decreased activity of SOD, CAT, or GPx [108, 109].

Conversely, the results of several studies implicated antioxidative effects of MTX. Zimmerman et al. showed that MTX inhibits the generation of MAA-protein adducts, formed by MDA and acetaldehyde interaction [110]. MAA-protein adducts have high immunogenic and prooxidative potential, so they are not only markers of oxidative stress, but also play an active role in the development of immune disorders. MTX significantly reduced the production of MAA-protein adducts as well as the level of free radicals *in vitro*. The reduction of free radicals and antioxidative action of MTX was achieved by the ability of MTX to directly scavenge O_2_^−^. Another study on the effects of 24 weeks of MTX therapy on oxidative stress showed antioxidative properties of MTX in psoriatic patients [111]. The levels of MDA significantly decreased, while TAS markedly increased after MTX therapy in psoriatic patients. Comparing to healthy controls the oxidative stress was higher in psoriatic patients both at the beginning and at the end of the MTX therapy protocol.

**Figure 6:**
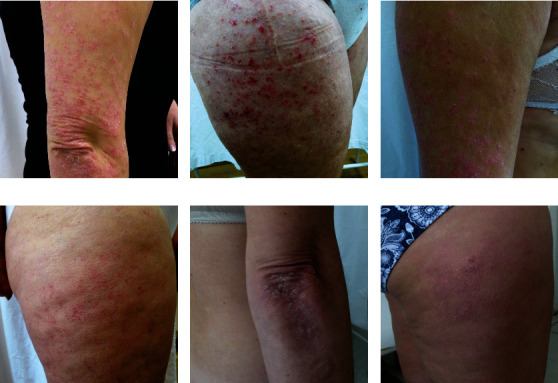
Clinical efficacy of methotrexate (MTX). Patient before starting therapy (a, b), after 8 weeks from the introduction of the MTX (15 mg per week) (c, d), and after 16 weeks after the introduction of the MTX (15 mg per week) (e, f). Pictures presented are part of the private collection of Vesna M. Milicic and Ana B. Ravic Nikolic.

Analyzing results of different studies, it can be concluded that MTX has somewhat contradictory impact on redox balance. This could be the consequence of its prooxidative and proapoptotic effects, and on the other hand, of decreased production of ROS through inhibition of inflammation-mediated pathways [112]. Clinical efficacy MTX is shown in Figure 6.

### 1.8. Effects of UVB and PUVA Therapy on Oxidative Stress

Phototherapy is often used in the treatment of skin diseases. It is a type of light therapy that mimics the effects of exposure to sunlight. Established phototherapies in psoriatic patients include PUVA radiation (psoralen in combination with UVA radiation) and narrow-band UVB (NB-UVB) [113]. The phototherapeutic procedures are usually effective in psoriasis treatment, but due to potential side-effects, including carcinogenesis, it can be used only for short-term treatment of the disease [113]. Increased ROS production during PUVA therapy, due to psoralen sensitization and UVA exposure, increases already increased levels of superoxide anion radical (O_2_^−^) and forms the basis of photocarcinogenesis [114].

Production of ROS due to PUVA therapy may cause oxidative damage to various macromolecules. The analysis of the PUVA effects on oxidative damage of DNA showed that suberythemal dose induced increased urinary extraction of 8-Oxo-2′-deoxyguanosine (8-oxo-dG) as a marker of DNA oxidation [115]. 8-oxo-dG in urine reached the peak 4 days after PUVA irradiation. *In vitro* study on HaCaT keratinocyte cell line also showed increased 8-oxo-dG production after exposure to therapeutic doses of PUVA and NB-UVB [116].

In the 12-week follow-up study, oxidative stress biomarkers, TBARS, and total antioxidant status (TAS) were significantly improved due to NB-UVB and PUVA therapy [117]. PUVA treatment appears to be more effective in the reduction of oxidative stress comparing to NB-UVB. The reduction of oxidative stress markers was connected with the reduction of inflammation. A study of similar design also showed the reduction of oxidative stress biomarkers, TBARS and TAS, after 12-week long PUVA and NB-UVB therapy [118]. Short-term increase of ROS production upon PUVA treatment, combined with the reduction of inflammation, could improve antioxidative potential and thus prolonged PUVA treatment results in the reduction of oxidative stress.

## 2. Conclusion

Oxidative stress and the aberrant immune response remain two crucial pathophysiological mechanisms in psoriasis. Their interdependence and interconnectedness represent the key to understanding etiopathogenesis of psoriasis. A large number of factors that can influence the development of psoriasis, from genetic predisposition to lifestyle, make the tangle of causes and consequences still insufficiently understood. Novel therapeutic options, primarily biological therapy, have significantly improved the quality of life of patients with psoriasis. However, the success of the applied therapy seems to depend on the reduction of both the proinflammatory and prooxidative components of the disease. The most noticeable and most powerful antioxidative effects exert various biologics compared to more convenient therapeutic modalities, such as methotrexate or phototherapy. Intersections of redox, immune, and inflammatory signaling pathways should be the focus of further researches dealing with pathophysiology of psoriasis, while antioxidative supplementation could be the solution in some refractory cases of the disease.

## Figures and Tables

**Table 1:** Biologics in psoriasis treatment.

Name of the drug	Biological target
Ustekinumab	IL-12 and IL-23—p40 subunit
Guselkumab	IL-23—p19 subunit
Tildrakizumab	
Risankizumab	
Secukinumab	IL-17
Ixekizumab	
Bimekizumab	
Brodalumab	IL-17 receptor
Etanercept	TNF-*α*
Infliximab	
Adalimumab	
Certolizumab pegol	
Golimumab	

**Table 2:** Main articles included in the summary of signaling cascades shown in Figure 2.

Article	Main findings
Marble et al. J Dermatol Sci. 2007; 48(2):87-101. [26]	CD11c + dendritic cells, CD68+ macrophages and TNF-*α* + cells are increased in psoriatic lesions
Ten Bergen et al. Scand J Immunol. 2020; 92(4):e12946. [27]	TNF-*α*/IL-23/IL-17 axis appears to has central role in the pathophysiology of psoriasis
Bos et al. Br J Dermatol. 2005; 152(6):1098-107. [28]	Activation of innate immunity, represented by the activity of NKT cells, dendritic cells, neutrophils, and keratinocytes, is crucial in pathogenesis of psoriasis.
Chiang et al. Front Immunol. 2019; 10 : 2376. [29]	The abundant presence of neutrophils in the psoriatic skin lesions and formation of Munro's microabscesses serves as a typical histopathologic hallmark of psoriasis.
Kim and Krueger. Dermatol Clin. 2015; 33(1):13-23. [13]	Keratinocytes appear to be important regulators of immune responses, involved in increased production of TNF-*α* or IFN-*γ* in psoriasis.
Hawkes et al. J Immunol. 2018; 201(6):1605-1613. [34]	Psoriasis is characterized by the presence of multiple T lymphocyte subsets (Th1, Th17, and Th22).
Stephen-Victor et al. PLoS Pathog. 2016; 12(6):e1005624 [37] Wang et al. J Cell Mol Med. 2019; 23(12):7926-7932. [38]	Th17 cells, besides IL-17, produce TNF-*α*, IL-26, and IL-29, which further stimulate release of proinflammatory mediators.
Levin and Gottlieb. J Am Acad Dermatol. 2014; 70(3):555-61. [46] Langrish et al. Immunol Rev. 2004; 202 : 96-105. [47]	IL-23 is recognized as a key regulator in psoriasis due to specific role in cross bridging the production of IL-17 by innate and acquired immunity.

**Table 3:** Papers included in the summary of signaling cascades shown in Figure 3.

Article	Main findings
Lai et al. Redox Rep. 2018; 23(1):130-135. [54]	ROS induces proliferation and differentiation of Th17/Th1/Th22 cells.
Johansen et al. Br J Dermatol. 2005; 152(1):37-42. [57]	Activity of the MAPKs and ERK1/2 is increased in psoriatic skin.
Müller et al. Autophagy. 2020; 16(8):1380-1395. [59]	ROS are important mediators in IL23A secretion via NF-*κ*B and MAPK pathways.
Zhou et al. Free Radic Biol Med. 2009; 47(7):891-905. [62]	Increased inflammation facilitates production of ROS and decreases already weakened antioxidative capacity
Srivastava et al. Indian J Dermatol Venereol Leprol. 2018; 84(1):39-44. [68]	Polymorphisms in the GST genes may result in increased production of ROS that could influence the pathogenesis of psoriasis.
Lee et al. J Invest Dermatol. 2013; 133(3):732-741. [70]	SOD deficiency resulted in more severe IL-23-mediated psoriasis-like skin inflammation.
Drewa et al. Med Sci Monit. 2002; 8(8):BR338-43. [71]	SOD and CAT activities were significantly lower in psoriatic patients.
